# Microsatellite Analysis of Five Populations of *Alosa braschnikowi* (Borodin, 1904) Across the Southern Coast of the Caspian Sea

**DOI:** 10.3389/fgene.2019.00760

**Published:** 2019-08-23

**Authors:** Omid Jafari, Jorge Manuel de Oliveira Fernandes, Ali-Akbar Hedayati, Ali Shabany, Maryam Nasrolahpourmoghadam

**Affiliations:** ^1^Department of Fisheries, Faculty of Fisheries and Environmental Sciences, Gorgan University of Agricultural Sciences and Natural Resources, Gorgan, Iran; ^2^Faculty of Biosciences and Aquaculture, Nord University, Bodø, Norway; ^3^Department of Fisheries Sciences, Faculty of Natural Resources, University of Tehran, Karaj, Iran

**Keywords:** allelic richness, *Alosa braschnikowi*, genetic diversity, population structure, SSR markers

## Abstract

Genetic diversity studies are essential in characterization of populations and species conservation. *Alosa braschnikowi* is a commercially valuable species native to the Caspian Sea. It is thought to have eight to nine subspecies, but the genetics of these populations remains to be investigated. The present study was performed to evaluate the genetic population structures of Caspian marine shad (*Alosa braschnikowi*) in the southern coast of the Caspian Sea using six pairs of SSR markers. A total of *Alosa braschnikowi* 140 specimens through five locations across the southern coast of the Caspian Sea were genotyped and 130 alleles were identified. The overall mean values of Ho and He were 0.58 and 0.87, respectively, with the highest and minimum value of Ho observed in Sari (0.67 ± 0.08) and Miankaleh (0.50 ± 0.04), respectively. The overall mean value of allelic richness was 12.6. The data suggest that there was a high rate of migration between populations of *Alosa braschnikowi* (overall mean of Nm = 13.57), with the highest value (19.07) between Gomishan and Mahmodabad locations. AMOVA results showed that 96% of variation was related to within populations and only 4% belonged to between populations. The mean Fst value of 0.019 indicates a low level of population differentiation. Our data suggest that there may be two genetically separate populations of *Alosa braschnikowi* across the southern coast of the Caspian Sea and a high rate of migration is likely to limit genetic diversity between them.

## Introduction

Genetic diversity is one of the basic prerequisites for the preservation and survival of a species and its adaptation to habitats subjected to different environmental pressures. It is thought that high genetic diversity improves the competence of individuals and increases the probability of species survival (Zoller et al., 1999; Hinten et al., 2003; Diz and Presa, 2009). Genetic diversity, namely, the difference in the number and type of alleles in the chromosomal loci, is crucial in stock assessment plans (Waldman and Yammarino, 1999). There are several markers that have been used to assess the genetic structure of populations and among them microsatellites or simple sequence repeats (SSRs) are one of the most commonly used markers (Crooijmans et al., 1997; Li et al., 2007). In recent years, several species and populations have been endangered as a result of overfishing and loss of nursery grounds in the Caspian Sea (Kiabi et al., 1999). These factors have a significant effect on genetic diversity reduction and homogenization of populations (McQuown et al., 2000).

Clupeidae is one of the most important fish family widely distributed in the Caspian Sea (Patimar et al., 2011). In the southern Caspian Sea, this family includes two genera: *Clupeonella* and *Alosa*. The latter consists of four species (Mousavi-Sabet et al., 2016), namely, *A. braschnikowi* (Borodin, 1904), A. caspia caspia (Eichwald, 1838), *A. kessleri* (Grimm, 1887), and *A. saposchnikowii* (Grimm, 1887). *Alosa braschnikowi* and *A. caspia* are thought to be native to the Caspian Sea and are more distributed in its southern areas. *A. braschnikowi* has a herring-like body shape, typically reaching 30 to 50 cm, and it usually feeds on small fishes [e.g., *Clupeonella engrauliformis* and gobies (*Neogobius*)] and also crustaceans (Coad, 2013). This species migrates within the Caspian Sea but never enters its tributary rivers. The high number of reported subspecies (up to nine) of *A. braschnikowi* may be an indication of population diversity, but this is likely affected by hybridization events between *A. braschnikowi* and other *Alosa* species and also between the various subspecies. In spite of the high frequency of this species in the Caspian Sea, there is a lack of molecular data and a modern revision is required to characterize its populations and ascertain their conservation status. It is well documented that for a successful conservation and effective management of a species, like designing a strategy of maintaining genetic diversity, it is highly essential to determine both genetic population structure and genetic variation within among populations (Sun et al., 2011). Hence, the present study was performed to determine the genetic population structure of *A. braschnikowi* across the southern regions of the Caspian Sea.

## Materials and Methods

### Sampling and DNA Extraction

**Figure 1:**
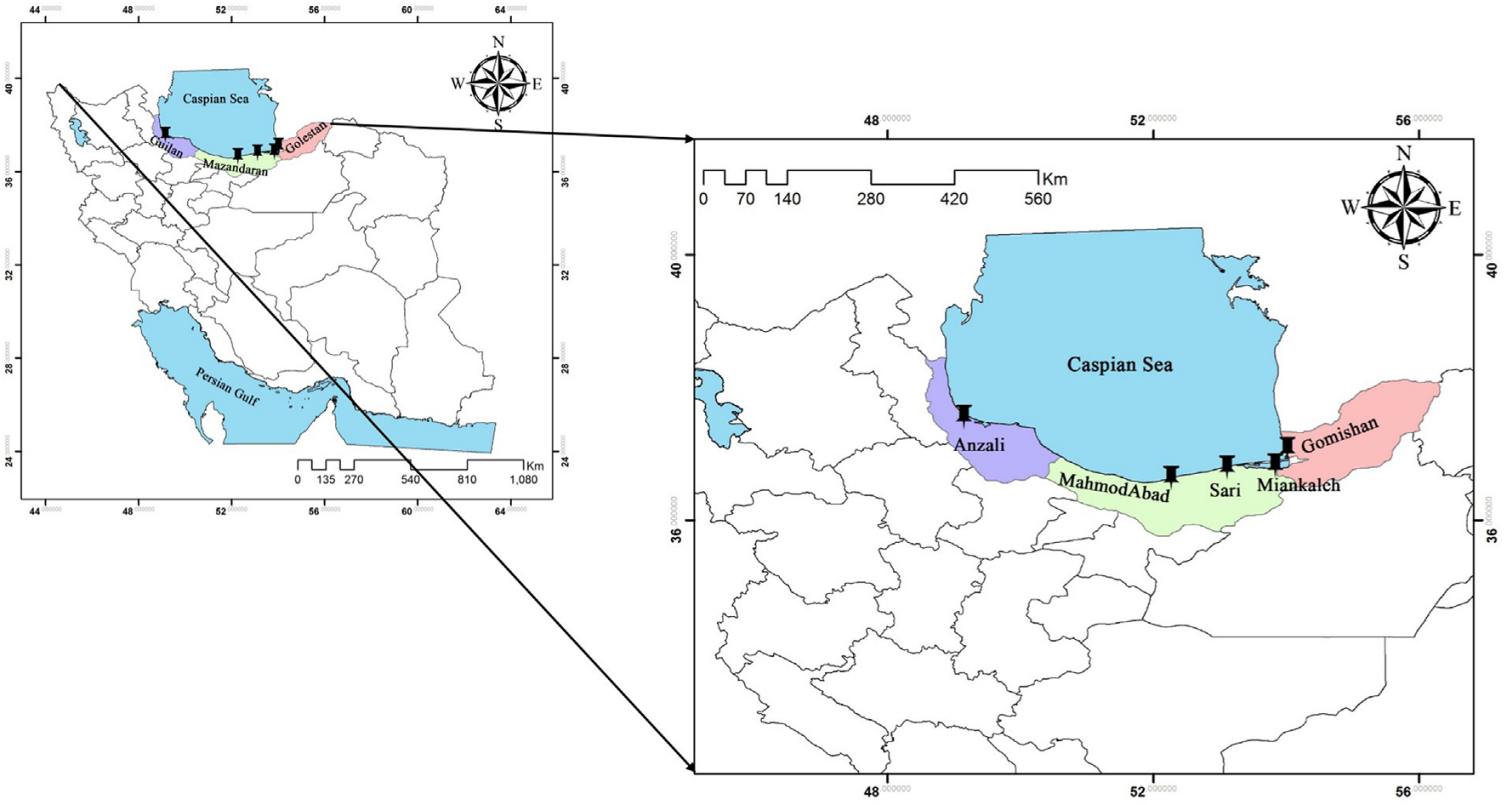
Sampling locations of *A. braschnikowi* across the southern coast of the Caspian Sea in this study.

In December 2013, a total of 140 specimens of *A. braschnikowi* (28 individuals per location) were sampled in five locations across the southern Caspian Sea including Anzali (E: 49°26’, N: 37°25’), Gomishan (E: 54°04’, N: 37°04’), Mahmood-Abad (E: 52°15’, N: 36°37’), Miankaleh (E: 53°35’, N: 36°48’), and Sari (E: 53°03’, N: 36°33’) (**Figure 1**), and sacrificed with an overdose of tricaine methanesulfonate (Pharmaq Ltd., UK) at 300 mg/L for 5 min. The dorsal fin of each fish was then clipped and stored in absolute ethanol until DNA extraction. DNA extraction was performed using DNeasy Blood & Tissue Kit (Qiagen, Valencia, CA, USA) following the manufacturer’s protocol. DNA integrity was checked by electrophoresis on a 1% (w/v) agarose gel and its concentration was determined using the NanoDrop (ND™-1000, Thermo Fisher Scientific, USA).

### Amplification of Microsatellites

Six pairs of microsatellite markers (AsaD030, AsaD042, AsaC051, AsaC059, AsaD312, and AsaD392) with high allele count and polymorphism were selected from data on *Alosa sapidissima* (Julian and Bartron, 2007) (**Table 1**). Polymerase chain reaction was performed in an MJ Mini Thermal Cycler (Bio-Rad, USA) with 25 μl PCR-specific microtubes containing 30 ng DNA (2 μl), 0.5 μl of each primer (10 pmol/μl), 1 μl of 10 mM dNTPs, 0.2 μl of Taq DNA polymerase (Fermentas, Thermo Fisher, Lithuania), 2.25 μl of 10× PCR buffer (Fermentas, Thermo Fisher, Lithuania), 1 μl of 50 mM MgCl_2_, and sterile distilled water. The amplification steps were as follows: In the first step, denaturation at 94°C for 5 min, followed by 35 cycles of 45 s at 94°C, annealing at the selected temperature (**Table 1**) for 30 s and extension at 72°C for 45 s, with a final extension for 5 min at 72°C. The PCR products were then visualized by electrophoresis on a 6% (w/v) acrylamide gel. A 50-bp DNA ladder (Fermentas) was used as a benchmark for determining the size of the alleles. The gels were stained using the silver-nitrate method (Bassam et al., 1991) and the Gel Pro analyzer package 3.9 (Gene, USA) was used to calculate the SSR lengths.

**Table 1:** SSR loci used on *A. braschnikowi* and their features. Primer sequences and annealing temperatures are also indicated.

Locus	GenBank accession no.	Number of alleles	Allele size	Primer sequence (5′→3′)	Annealing *T* (˚C)
AsaD030	EF014998	13	104–152	F: CCACAGCATCATCTCTTTACTG R: ACCTTGAATTTCTCCTTGGG	55
AsaD042	EF015000	17	124–192	F:ACTGGTCAATTGTAAGACACCC R:CAAGATGACCAAGGGTTAAGAC	50
AsaC051	EF014992	27	132–192	F: GTAAGTCGCTTTGGACTACCAG R:TCTAAATGCCCAGGTAAAGATG	53
AsaC059	EF014993	26	288–392	F: CTTGGACTTACAATGCTTTTGG R: AGCAAGTGTGGAGTCAGTCG	53
AsaD312	EF014999	16	132–192	F: TAAACATACTGCTCCTTCACCC R: ATGTGCTCTTGTTTCAATGATG	54
AsaD392	EF015004	30	128–280	F:ATGATGTAAAACCAGGAGATGC R: CATAGGTCTTAAAACGTGGGTG	53

### Statistical Analysis

Observed heterozygosity (Ho), expected heterozygosity (He), and deviation from Hardy–Weinberg equilibrium (HWE) were determined using GenAlex 6.5 (Peakall and Smouse, 2006). Errors in allele scoring, large allele dropout, and null alleles were investigated with Micro-checker 2.2.1 (Van Oosterhout et al., 2004). A non-parametric Wilcoxon test (Zar, 1999) in SPSS software Ver 20 was used to determine the level of difference between Ho and He values. Using FSTAT software (ver 2.9.3), values of allele richness and Fis were determined (Goudet, 2002). Among and between populations, genetic diversity and separation values between locations based on Fst index were obtained by AMOVA using GenAlex 6.41 (Peakall and Smouse, 2006).

A linkage disequilibrium test between pairs of genetic sites was done using the GENEPOP 4.0.10 software (Rousset, 2008). Unbiased genetic distances (D) and genetic identities (I) according to Nei (Nei, 1978) were calculated in Popgene 1.0. A Bayesian approach in STRUCTURE v.2.3.4 was used to deduce the population structure of *A. braschnikowi* and to make an estimation of genetically detached populations (Pritchard et al., 2000). The number of populations (*K*) was estimated considering 10,000 as length of burn-in period and 100,000 of MCMC reps after burn-in, with an assumption of *K* = 1–5 and 100 iterations. The best *K* was decided based on the Delta *K* method suggested by Evanno et al (2005). The Bottleneck software 1.2.02 was used to determine the probability of recent bottleneck events with 1,000 iterations based on either heterozygosity excess or deficiency (Cornuet and Luikart, 1996).

## Results

### Allele Identification

All SSR markers used in this study showed a high level of polymorphism in *A. braschnikowi* (**Table 1**). A total of 1,680 fragments were amplified from five populations of *A. braschnikowi* using six SSR markers ranging from 104 to 392 nt in length. The 130 alleles identified were distributed in the range 13–30 per SSR locus. In all populations, there were 16 alleles through all loci that were unique or private alleles (**Supplementary Table S1**). Total average of observed alleles through all loci was 12.6; its range was 7–20 alleles, belonging to Sari and Miankaleh locations, respectively (**Table 2**).

**Table 2:** Polymorphic information at six SSR loci of five wild populations of *A. braschnikowi*.

Locus	Anzali					Gomishan					Mahmodabad					Miankaleh					Sari				
	Na	Ne	Ho	He	Fis	Na	Ne	Ho	He	Fis	Na	Ne	Ho	He	Fis	Na	Ne	Ho	He	Fis	Na	Ne	Ho	He	Fis
AsaD030	8	5.15	0.75	0.81	0.08^**^	12	7.64	0.68	0.87	0.23^**^	10	7.29	0.71	0.86	0.19	8	4.68	0.50	0.78	0.38	7	4.96	0.86	0.79	−0.05
AsaD042	10	7.64	0.32	0.87	0.64^**^	9	5.54	0.28	0.82	0.66^**^	10	6.90	0.53	0.85	0.38^**^	10	6.47	0.28	0.84	0.67^**^	15	11.52	0.57	0.91	0.39^**^
AsaC051	15	11.12	0.50	0.91	0.46^**^	16	11.12	0.64	0.91	0.31^**^	15	10.66	0.71	0.90	0.23^**^	16	8.66	0.50	0.88	0.45^**^	17	11.96	0.75	0.92	0.19^**^
AsaC059	8	4.99	0.25	0.80	0.69^**^	11	7.53	0.21	0.87	0.76^**^	17	9.73	0.35	0.89	0.61^**^	20	10.45	0.57	0.90	0.38^**^	13	8.04	0.35	0.87	0.60^**^
AsaD312	12	7.80	0.64	0.87	0.27^**^	11	7.91	0.43	0.87	0.52^**^	10	8.16	0.82	0.88	0.08^**^	10	7.76	0.50	0.87	0.44^**^	12	7.49	0.54	0.86	0.39^**^
AsaD392	16	9.73	0.68	0.90	0.26^**^	16	10.88	0.85	0.91	0.07	18	11.20	0.68	0.91	0.27^*^	12	8.71	0.64	0.88	0.29^**^	15	11.52	0.89	0.91	0.04^**^
Average	11.5	8.21	0.53	0.86	0.40	12.5	8.65	0.52	0.87	0.42	13.33	8.82	0.64	0.88	0.29	12.67	7.56	0.50	0.86	0.43	13.16	9.07	0.66	0.88	0.26

Na, number of alleles; Ne, number of effective alleles; Ho, observed heterozygosity; He, expected heterozygosity; Fis, inbreeding coefficient. ** Significant deviation from HWE at P < 0.05.

### Linkage Analysis and Population Genetic Variation

The Genepop genotypic linkage disequilibrium analysis at each locus in each population revealed that there were no loci out of equilibrium in all populations (*P* > 0.05).

The average number of effective alleles in Anazali, Gomishan, Mahmodabad, Miankaleh, and Sari were 8.2, 8.65, 8.82, 7.56, and 9.07, respectively. Microchecker did not show any evidence of large allele dropout and scoring error but there was a probability of 0.24 by null alleles. The highest (0.89) and lowest (0.21) values of Ho were found at loci AsaD392 (Sari location) and AsaC059 (Gomishan area), respectively (**Table 2**). After Bonferroni correction, there was a high rate of deviation from HWE. The total mean of observed heterozygosity was 0.58 with maximum and minimum values of 0.67 and 0.50 in Sari and Miankaleh, respectively (**Table 3**). Also, the overall mean value of He was 0.87. The total mean value of inbreeding coefficient (Fis) at loci was 0.34, with maximum and minimum values of 0.43 and 0.26 in Miankaleh and Sari, respectively (**Table 3**). The maximum value (0.94) of polymorphic information content (PIC) was observed at loci AsaD392 and AsaC051, while the minimum value was obtained for AsaD030 (0.85). The PIC values for the all investigated loci are available in **Table 4**.

**Table 3:** Observed and expected values of heterozygosity and Fis index at different locations of *A. braschnikowi*. Mean values are also indicated.

	Anzali	Gomishan	Mahmodabad	Miankaleh	Sari	Mean ± S.D.
Ho	0.53 ± 0.08	0.55 ± 0.09	0.63 ± 0.07	0.50 ± 0.04	0.67 ± 0.08	0.58 ± 0.03
He	0.85 ± 0.01	0.87 ± 0.01	0.88 ± 0.01	0.86 ± 0.01	0.88 ± 0.01	0.87 ± 0.007
Fis	0.406	0.423	0.297	0.435	0.266	0.34 ± 0.07

**Table 4:** Polymorphism information content (PIC) values of the SSR markers examined in *A. braschnikowi*.

Locus	AsaD030	AsaD042	AsaC051	AsaC059	AsaD312	AsaD392
PIC	0.85	0.88	0.94	0.92	0.90	0.94

### Population Genetic Differentiation and Cluster Analysis

**Figure 2:**
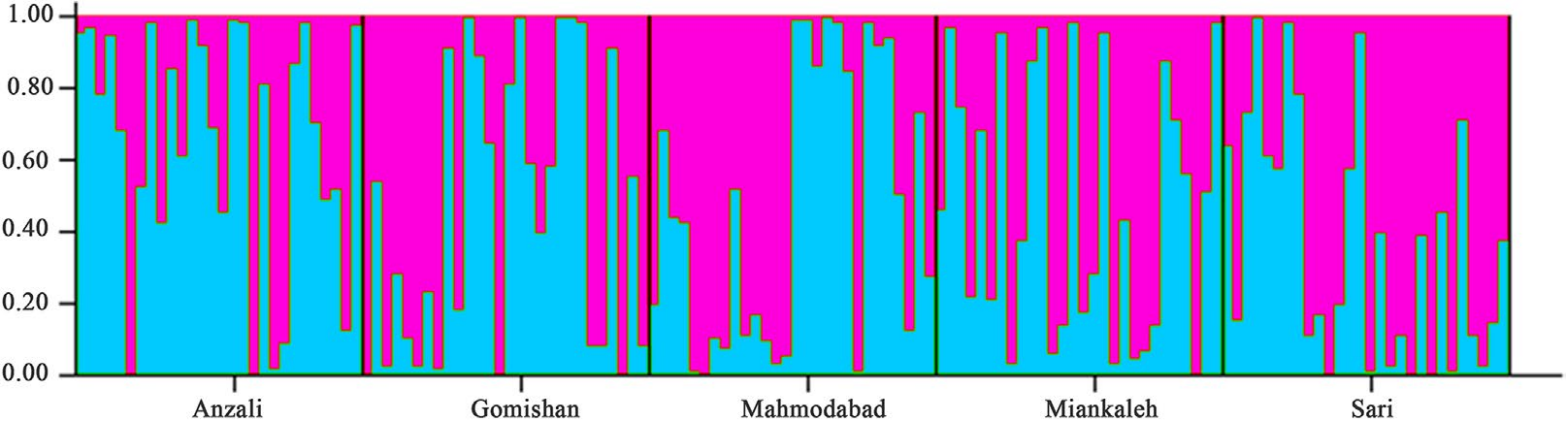
Admixture analysis of *A. braschnikowi* at *K* = 2; each bar represents an individual and the Y axis shows the probability of individuals belonging to the identified populations. For each individual, the different colors are related to the number of markers shared with the other cluster.

**Figure 3:**
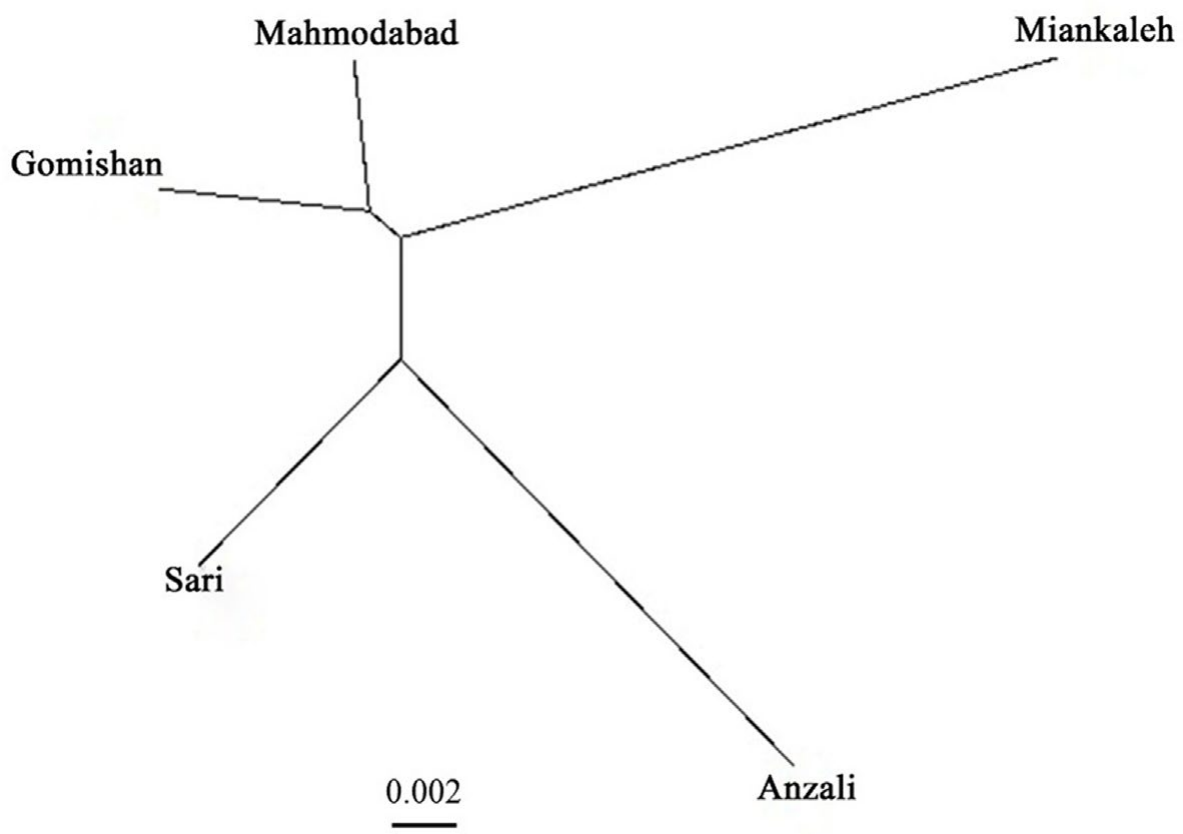
Bayesian tree of *A. braschnikowi* populations from five locations in the Caspian Sea. The length of the branches reflects the net nucleotide distance between groups.

The total mean value of Fst was 0.019 and the AMOVA results revealed that it was mostly related to within population variation (96%) rather than variation among populations (4%). Average gene flow (Nm) was calculated as 13.57 and the highest rate of Nm (19.07) was observed between Gomishan and Mahmodabad (**Table 5**). All Fst values between populations are also presented in **Table 5**, with the highest value observed between Anzali and Miankaleh locations (Fst = 0.025). Genetic identity and genetic distance parameters based on Nei index are shown on **Table 6**. The maximum and minimum genetic distances were observed between Anzali and Miankaleh (0.273) and Gomishan and Mahmodabad (0.07) locations, respectively. The Mantel test also revealed that there was no significant correlation between genetic and geographical distances (*R* ^2^ = 0.25, P = 0.12). SSR cluster analysis revealed that the best *K* fitting to the data was 2, indicating genetically segregate populations [Mean LnP(*K*) = −4,116.400000, Stdev LnP(*K*) = 2.722132, Delta *K* = 11.417523] (**Figure 2**). The Bayesian tree based on allele frequency divergence (net nucleotide distance) comprised two main clades: i) Anzali and Sari, and ii) Gomishan, Mahmodabad, and Miankaleh (**Figure 3**). The net nucleotide distances between locations are provided in **Supplementary Table S2**. Also, results from Bottleneck analysis did not show any evidence for genetic bottleneck in any A. braschnikowi population (p of H excess or deficiency = 0.11).

**Table 5:** Fst (below diagonal) and Nm (above diagonal) values between *A. braschnikowi* from different locations.

Locations	Anzali	Gomishan	Mahmodabad	Miankaleh	Sari
Anzali	0.000	15.93	13.92	9.63	15.38
Gomishan	0.015	0.000	19.07	14.16	13.11
Mahmodabad	0.018	0.013	0.000	10.89	12.34
Miankaleh	0.025	0.017	0.022	0.000	11.22
Sari	0.016	0.019	0.020	0.022	0.000

**Table 6:** Matrix of genetic distances (above diagonal) and identities (below diagonal) between different locations of *A. braschnikowi*.

Locations	Anzali	Gomishan	Mahmodabad	Miankaleh	Sari
Anzali	−	0.103	0.142	0.273	0.115
Gomishan	0.903	−	0.070	0.132	0.181
Mahmodabad	0.867	0.933	−	0.233	0.212
Miankaleh	0.761	0.876	0.792	−	0.221
Sari	0.891	0.834	0.809	0.802	−

## Discussion

Genetic diversity in the structure of populations is one of the most important and essential principles for the survival and development of organisms (Diz and Presa, 2009). Overfishing (Bergh and Getz, 1989) and some other environmental stressors such as pollution and loss of habitat (Dudgeon et al., 2006) decrease the ability of a population to maintain its genetic diversity by reducing the effective population size. In genetic population studies, various parameters such as heterozygosity and the number of alleles (factual and effective) are used. At present, there are no specific SSR markers for the Alosa genus in the Caspian Sea. The usefulness of SSR markers depends on their polymorphism information content, which reflects the ability of a marker to detect a polymorphism in a given population (Dudu et al., 2015). We have demonstrated that the six SSRs found in American shad (*Alosa sapidissima*) (Julian and Bartron, 2007) show a high rate of polymorphism (PIC = 0.90) in *A. braschnikowi* as well. Heterozygosity as an index of genetic diversity shows the proportion of heterozygous loci in a given population (García‐Navas et al., 2014). The overall mean of observed heterozygosity at the population level was 0.58, which is higher than the reported value for freshwater fish (0.54 ± 0.25). Rare alleles have a low abundance (less than 0.01); thus, they have a minor contribution to heterozygosity and their removal does not have much effect on the observed heterozygosity. Because of this, in genetic population studies, this parameter (heterozygosity) alone cannot provide reliable results, which are mostly based on random variation in allele frequency (Kitada et al., 2009). Also, allelic variation of heterozygosity is more important in population genetic studies, and its increase or decrease can reflect the changes in the effective population size. Allelic richness is a more useful parameter in genetic population studies rather than heterozygosity (Li et al., 2009). Effective allele is a very important indicator in population studies and its increase or decrease can reflect the increase or decrease of the effective size of the population (Diz and Presa, 2009; Li et al., 2009). In the present study, based on the results from allelic richness analysis, the overall mean value of 12.61 alleles was obtained at the population level, which is also more than the reference value for freshwater fish (9.1 ± 6.1); also, all loci examined in this study showed values close to or higher than those reported for freshwater fish (AsaD030 = 9.0, AsaD042 = 10.8, AsaC051 = 16.0, AsaC059 = 13.8, AsaD312 = 11.2, AsaD392 = 15.8) (DeWoody and Avise, 2000). Unfortunately, there are no reports in Iranian *Alosa* sp. to enable a direct comparison. Nevertheless, the genetic diversity indices (Na = 15.4 and Ho = 0.81) in *A. sapidissima* (Julian and Bartron, 2007) are higher compared to the *A. braschnikowi* in this study, which may be related to the different number of specimens and markers used. In another study using eight pairs of dinucleotide microsatellite markers on *Alosa fallax* and *A. alosa*, the mean alleles per locus were 4.50 and 4.88, respectively (Faria et al., 2004). The level of heterozygosity in *A. fallax* and *A. alosa* species was 0.560 and 0.444, respectively. Differences in species type, primers, and sample size restrict direct comparisons, but our results suggest a relatively higher genetic variation of the *A. braschnikowi* compared to *A. fallax* and *A. alosa*.

The observed heterozygosity is significantly less than the expected value in *A. braschnikowi* (*P* < 0.05). There are several reasons that can explain this significant reduction in Ho compared with He, namely, high rate of migration, errors in reading alleles, and inbreeding (Skaala et al., 2004; Li et al., 2009). The obtained results of inbreeding coefficient showed a significant heterozygosity deficit through the loci (*P* = 0.0016). Also, there was a high rate of deviation from HWE (26 out of 30), which can be because of mixing between populations (natural migration) with different allele frequencies (Wahlund effect) (Karlsson and Mork, 2005). Based on the obtained Fst values, it can be concluded that the rate of differentiation between populations is low but significant (mean overall Fst value = 0.019, *P* = 0.01) (Balloux and Lugon-Moulin, 2002) and a high rate of migration can be the main factor underlying this low differentiation. Moreover, the patterns of genetic variation among *A. braschnikowi* populations suggest at least a historical connectivity between these now geographically distant populations, similarly to what has been observed in empire gudgeon, *Hypseleotris compressa* (McGlashan and Hughes, 2001). Another possible reason for low differentiation is the high rate of genetic diversity within each population, as we observed that 96% of variation is related to the within-population diversity. The gene flow between populations and individuals with very different allele frequencies increases the genetic variation within a population but tends to make different populations genetically similar to each other. A bottleneck analysis revealed that the *A. braschnikowi* populations studied do show signs of genetic bottlenecks. Hence, the more likely explanations for the limited differentiation observed among these Caspian *A. braschnikowi* populations are i) gene flow and/or ii) the lack of isolation for enough time to enable genetic drift and selection to cause a noticeable differentiation.

*A. braschnikowi* is known to exhibit migratory behavior through the Caspian Sea but there is a basic need to closely monitor them to avoid the loss of *Alosa* stocks in Iran. Also, it seems that there are at least two genetically different stocks of *A. braschnikowi* in the southern coast of the Caspian Sea: one clade including fish from Anzali and Sari locations (South to Southwest), and the second one comprising Gomishan, Mahmodabad and Miankaleh locations (South to Southeast). Based on Thorpe (1982), populations belonging to same species and same genus have genetic identity in the range 0.80 to 0.97 and 0.35 to 0.85, respectively. The genetic identity within *A. braschnikowi* in the present study was 0.76 to 0.93, indicating that all populations studied indeed belong to same species. The lower observed values of genetic identity may be due to a lower gene flow between some locations like Miankaleh and Anzali, suggesting that they may be different subspecies. *A. brascnikowi* subspecies have been recently classified based on phenotypic traits, such as the number of gill rakers and diet (Coad, 2013). Integrating morphologic and genetic data will be an effective way to improve the classification of *A. braschnikowi* subspecies and also to determine if the populations examined in the present study correspond to any of the subspecies already identified using phenotypic characters.

To the best of our knowledge, this is the first molecular population study performed on *A. braschnikowi* from the southern Caspian Sea. Our data indicated that there are at least two genetically distinct subpopulations of *A. braschnikowi* in this area. It seems that *A. braschnikowi* harbor high genetic diversity in both terms of observed heterozygosity and allelic richness across the southern coast of the Caspian Sea. The obtained results of this study accompanied by some other biological data like morphology and mtDNA analysis can aid in the conservation of this species, since its current status is undetermined because of data deficiency (Di Dario, 2017).

## Data Availability

All datasets generated for this study are included in the manuscript and the supplementary files.

## Ethics Statement

This study was carried out in accordance with the recommendations of the Canadian Council on Animal Care, as implemented by the ethics committee of Gorgan University of Agricultural Sciences and Natural Resources (Iran). The protocol was approved by the ethics committee of Gorgan university of Agricultural Sciences and Natural Resources.

## Author Contributions

OJ designed and performed the experiments, analyzed the data, and wrote the manuscript. JF contributed to data analysis and reviewed the manuscript. MP assisted in sampling and laboratory experiments. A-AH contributed to writing the manuscript, and AS supervised the work and provided the required laboratory infrastructure.

## Funding

The authors are grateful to Gorgan University of Agricultural Sciences and Natural Resources (Iran) for providing the required financial, technical and administrative support, and to Nord University (Norway) for covering the open access publication fee.

## Conflict of Interest Statement

The authors declare that the research was conducted in the absence of any commercial or financial relationships that could be construed as a potential conflict of interest.
