# Supplementary material for: Microsatellite Analysis of Five Populations of Alosa braschnikowi (Borodin, 1904) Across the Southern Coast of the Caspian Sea
Source: Front Genet. 2019 Aug 23;10:760. doi: 10.3389/fgene.2019.00760 (PMC6736623; doi:10.3389/fgene.2019.00760)
Supplement: Supplementary file 1 [file DataSheet_1.pdf]

Supplementary Table S1. Private allele information based on SSR markers in *A. braschnikowi*.

Sample No.	Population	Loci with Private Alleles
20	Anzali	AsaD312
2	Gomishan	AsaD030
5	Gomishan	AsaD030
13	Miankaleh	AsaC059
14	Miankaleh	AsaC059
15	Miankaleh	AsaC059
16	Miankaleh	AsaC059
1	Sari	AsaD042
2	Sari	AsaD042
3	Sari	AsaD042
4	Sari	AsaD042
5	Sari	AsaD042
6	Sari	AsaD042
7	Sari	AsaD042
8	Sari	AsaD042
20	Sari	AsaD042
