# Supplementary material for: Microsatellite Analysis of Five Populations of Alosa braschnikowi (Borodin, 1904) Across the Southern Coast of the Caspian Sea
Source: Front Genet. 2019 Aug 23;10:760. doi: 10.3389/fgene.2019.00760 (PMC6736623; doi:10.3389/fgene.2019.00760)
Supplement: Supplementary file 2 [file DataSheet_2.pdf]

Table S2. Allele-frequency divergence among populations (net nucleotide distance).

	Anzali	Gomishan	Mahmodabad	Miankaleh	Sari
Anzali	0.00	0.0410	0.0427	0.0554	0.0380
Gomishan		0.00	0.0155	0.0373	0.0263
Mahmodabad			0.00	0.0345	0.0284
Miankaleh				0.00	0.0473
Sari					0.00
